# Effect of Cadmium and Copper Exposure on Growth, Secondary Metabolites and Antioxidant Activity in the Medicinal Plant Sambung Nyawa (*Gynura procumbens* (Lour.) Merr)

**DOI:** 10.3390/molecules22101623

**Published:** 2017-10-12

**Authors:** Mohd Hafiz Ibrahim, Yap Chee Kong, Nurul Amalina Mohd Zain

**Affiliations:** 1 Department of Biology, Faculty of Science, Universiti Putra Malaysia, UPM Serdang, Selangor Darul Ehsan 43400, Malaysia; yapchee@upm.edu.my; 2 Department of Biology, Institute of Biological Science, Faculty of Science, University Malaya, Kuala Lumpur 50603, Malaysia

**Keywords:** medicinal plants, heavy metal contamination, secondary metabolites, antibacterial activity

## Abstract

A randomized complete block (RCBD) study was designed to investigate the effects of cadmium (Cd) and copper (Cu) on the growth, bioaccumulation of the two heavy metals, metabolite content and antibacterial activities in *Gyanura procumbens* (Lour.) Merr. Nine treatments including (1) control (no Cd and Cu); (2) Cd 2 = cadmium 2 mg/L; (3) Cd 4 = cadmium 4 mg/L; (4) Cu 70 = copper 70 mg/L; (5) Cu 140 = copper 140 mg/L); (6) Cd 2 + Cu 70 = cadmium 2 mg/L + copper 70 mg/L); (7) Cd 2 + Cu 140 = cadmium 2 mg/L + copper 70 mg/L); (8) Cd 4 + Cu 70 = cadmium 4 mg/L+ copper 70 mg/L and (9) Cd 4 + Cu 140 = cadmium 4 mg/L + copper 140 mg/L) were evaluated in this experiment. It was found that the growth parameters (plant dry weight, total leaf area and basal diameter) were reduced with the exposure to increased concentrations of Cd and Cu and further decreased under interaction between Cd and Cu. Production of total phenolics, flavonoids and saponin was observed to be reduced under combined Cd and Cu treatment. The reduction in the production of plant secondary metabolites might be due to lower phenyl alanine lyase (PAL) activity under these conditions. Due to that, the 1,1-diphenyl-2-picrylhydrazyl (DPPH), ferric reducing antioxidant potential (FRAP) and antibacterial activities was also found to be reduced by the combined treatments. The current experiments show that the medicinal properties of *G. procumbens* are reduced by cadmium and copper contamination. The accumulation of heavy metal also was found to be higher than the safety level recommended by the WHO in the single and combined treatments of Cd and Cu. These results indicate that exposure of *G. procumbens* to Cd and Cu contaminated soil may potentially harm consumers due to bioaccumulation of metals and reduced efficacy of the herbal product.

## 1. Introduction

Due to human activities, the content of heavy metals in soils keeps increasing due to increased industrialization, energy production, agriculture activities and municipal waste production [1]. This has led to an accumulation of non-essential and toxic heavy metals such as aluminium (Al), arsenic (As), cadmium (Cd), manganese (Mn) and chromium (Cr) in Malaysian agricultural soils. Although these trace elements are important for plant growth (essential micronutrients), others have no metabolic role and may adversely affect plant growth and metabolism [2]. High concentrations of these metals might cause growth inhibition and even plant death. These heavy metals have the capability to be transferred and accumulated in plants, animals and humans [3]. Moreover, these heavy metals have long term persistence in the human body, which might cause hazardous health issues to humans. There are several factors influencing the concentrations of these metals in plants such as the plant species, microclimate conditions, environmental pollution and other factors [4]. Among these heavy metals, cadmium (Cd^2+^) and copper (Cu^2+)^ are known to delay plant growth and cause the formation of reactive oxygen species and thus affect membrane function and permeability [5].

Cadmium is one of the heavy metals that has a high impact on environment degradation. The contamination with this heavy metal is due to the manufacture of diverse products such as batteries, chipsets, pigments, television receivers, and semiconductors [6]. Cadmium is easily taken up by plant roots and transported to the aerial parts, thus entering the food chain and causing health problems in animals and humans. Cadmium can easily bind to sulfated groups, e.g., as found in metalloproteins and metalloenzymes, thereby degrading their functions [7]. Cadmium also does not have any benefit or nutritional value for plants, and excessive exposure to this heavy metal can cause toxicity indicated by altered chloroplast ultrastructure, chlorosis-stunted plant growth, reduction in photosynthesis, degradation of enzymes involved in CO_2_ fixation, lipid peroxidation and disturbance of the nitrogen (N) and sulfur (S) metabolism [8].

Copper is an essential element for plant growth and development, but because only a small amount is needed, it is classified as a micronutrient. The amount of Cu available to plants varies widely by soils. Available Cu can vary from 1 to 200 ppm (parts per million) in both mineral and organic soils as a function of soil pH and soil texture [9]. Several sources of Cu contamination exist in the environment, including industrial and domestic waste, agricultural practices, copper marine drainage, copper-based pesticides, and antifouling paints. This metal is a structural element in regulatory proteins, photosynthetic electron transport, mitochondrial respiration, oxidative stress, cell wall metabolism, transcription, protein trafficking, and hormone signalling [10]. However, in excess, it can inhibit growth and impair important cellular processes like photosynthetic electron transport, photosynthesis, and respiration. Membrane transport systems seem to be a target of this metal, playing a central role in its toxicity processes [11].

Exposure to heavy metals leads to accumulation of harmful reactive oxygen species (ROS). Plants counteract the toxic effects of heavy metal stress by activating certain metabolic activities and physiological modifications [12]. These protect the plant against free radicals and prevent damage to plant molecules such as lipids, proteins and nucleic acids. Some of the strategies include accumulation of plant secondary metabolites such as antioxidant enzymes, proline, glutathione and phenolic and flavonoids compounds. These plant secondary metabolites play numerous roles in plant protection under different biotic and abiotic influences [13,14]. Phenolic compounds and other secondary metabolites have numerous pharmacological properties and thus any environmental condition that affects either the quantity or composition of phytochemical compounds may potentially influence the efficacy of the medicinal plant product. There is limited information on specific physiological responses of medicinal plants to heavy metals in soils and the resulting changes in the efficacy of the plant [15,16].

*Gynura procumbens* which belongs to the botanical Compositae family is locally known in Malaysia as ‘Sambung nyawa’ [17]. This plant is about 15–30 cm high and has succulent, elliptic and glossy purplish leaves. The leaves of *G. procumbens* have been consumed as a salad for decades in Malaysia. A group of researchers has indicated the presence of multiple classes of compounds in *G. procumbens* extract such as alkaloids, coumarins, flavonoids, triterpenes, and valepotriates [18]. Due to its content of these secondary metabolites, the plant has been widely used as a traditional medicine to treat cancer, kidney disease, migraines, hypertension and diabetes [17,18,19]. Extracts of this plant have pharmacological activities such as anti-hyperglycemic, anti-inflammatory and anti-hypertensive effects [19]. Cultivation of this medicinal plant is gaining popularity with increasing government support to meet the high demand for plant materials. Currently, this herb is listed as a high value herbal plant under the Malaysia Agricultural National Key Economic Area Initiative for the herbal subsector (NKEA). Normally *G. procumbens* has been planted in beds for easy cultivation in nurseries. However, a recent study by Ong et al. [20] showed that most surface soils in Malaysia are contaminated with heavy metals, especially Cd and Cu. This suggested that there might be cadmium and copper contamination in the surface soil used for the preparation of plant beds [21]. There is a concern that these contaminants might be transferred to humans through the food chain [22]. Uptake and accumulation of heavy metals in medicinal plants not only poses a serious safety threat to consumers, but also affects the efficacy and quality of derived medicinal plant products [23,24]. Previously, no study has been conducted to look into the impact of the interactions of cadmium and copper with herbal medicines. This work will provide the basic understanding of heavy metal (Cd, Cu) relationship with herbal medicine in terms of growth, bioaccumulation, antioxidant and antimicrobial activities. Thus, the aim of the present study was to determine the effects of Cd, Cu and its combinations on the growth and heavy metal bioaccumulation in *G. procumbens*. In addition, total chlorophyll content was quantified as an indication of stress responses to heavy metals. The secondary metabolite content (total phenolics, flavonoids and saponin), antioxidant and antibacterial activity was determined to establish the effect of these heavy metal contamination on the efficacy of *G. procumbens*.

## 2. Results and Discussion

### 2.1. Growth Parameters

Table 1 shows the growth patterns of *G. procumbens* under Cd and Cu exposure. Generally, it was seen that plant dry weight, total leaf area and basal diameter decrease with increasing Cd treatments (*p* ≤ 0.05) in *G. procumbens*. The highest concentration of Cd (4 mg/L) decreased the plant dry weight, total leaf area and basal diameter compared to the control plants. Plant dry weight, total leaf area and basal diameter was highest under lowest Cu treatments (Cu 70) compared to the highest Cu treatments (Cu 140). In the current study, growth parameters of *G. procumbens* grown in the combined treatments (Cd + Cu) were statistically lower compared to Cd and Cu treated plants. The current results indicate that a combination of Cd and Cu would reduce growth parameters more compared to the application of Cd and Cu alone. 

Furthermore, in the correlation Table 2, the growth parameters (plant dry weight, total leaf area and basal diameter) have a significant negative correlation with cadmium and copper. This implies that presence of cadmium and copper would reduce the plant growth. This result was in agreement with work by Di Santo et al. [25] where they observed *Quercus pubescence* plantlets that were exposed to cadmium and copper had lower growth patterns compared to seedlings treated with cadmium and copper alone. In addition, Deng et al. [26] observed that combination of Cd and Cu reduced the growth patterns of germinated maize seeds. Furthermore, Versieran et al. [27] found that Cu and Cd combination was more toxic to barley seedlings compared to Zn and Cd as measured by inhibition of root elongation in barley seedlings. It can be concluded that growth of this herb on contaminated soils can slow the plant growth by reduction in dry weight accumulation, lower leaf area and reduce thickness of the plant stem.

### 2.2. Total Chlorophyll Content 

The impact of Cd and Cu on total chlorophyll content of G. *procumbens* is presented in Table 1. The total chlorophyll content decreased with increasing rates of Cd and Cu. This herb has higher levels of chlorophyll b than chlorophyll a. The result suggested that the decrease in total chlorophyll content in this plant likely to be due to degradation of chlorophyll synthesis under exposure to high levels of Cd and Cu. Chlorophyll content is often measured in plants in order to assess the impact of environmental stress, as changes in pigment content are linked to visual symptoms of plant illness and photosynthetic productivity [28]. Heavy metals inhibit metabolic processes by inhibiting the action of enzymes, and this may be the most important cause of chlorophyll degradation. Decreased chlorophyll content associated with heavy metal stress may be the result of inhibition of the enzymes responsible for chlorophyll biosynthesis [29]. In the present study, total chlorophyll content was found to be negatively correlated with cadmium (r^2^ = −0.765; *p* ≤ 0.05; Table 2). This suggests that cadmium might play the role in reduction of total chlorophyll content in the present study. This was supported by study by Tuna et al. [30] on maize that found among heavy metals such as lead, copper and mercury, cadmium had a more profound effect in reducing the TCC compared to the influence of other heavy metals. It can be concluded from the present study that exposure to heavy metals can reduce TCC content by reduction in chlorophyll a and b as observed in *G. procumbens*.

### 2.3. Uptake and Distribution of Cd and Cu in Plants

Root to shoot translocation of the two heavy metals in *G. procumbens* is shown in Table 3. The Cd concentration was lower in the shoot compared to the root. Similarly, the translocation of Cu also was poor (TF < 1). The highest level of Cu was observed in the roots compared to the shoots. The highest TF for Cd and Cu was observed in the combined treatment of Cd 2 + Cu 70. Accumulation of Cd and Cu was lower in the shoots compared to the roots, which indicated a slow translocation of these elements from the root to the shoot part. From the result, it also can be concluded that this plant was not a hyperaccumulator. Usually, a plant that accumulates high concentrations of Cd and Cu (>100 mg/kg and 1000 mg/kg respectively) in their tissue can be categorized as a hyperaccumulator [31]. Although this plant is not a hyperaccumulator, when exposed to the heavy metals in the present study, this plant could accumulate Cd and Cu above the WHO-recommended safety limits that are 0.3 mg/kg and 1.3 mg/kg, respectively [32]. At low doses, cadmium can directly induce toxic effects that affect mainly the human kidney and liver. Chronic exposure of the human body can induce a disease called itai–itai, in which patients show a wide range of symptoms such as low grades of bone mineralization, high rates of fractures, increased rates of osteoporosis, and intense bone-associated pain [30]. Ingestion of high levels of copper can cause copper poisoning that is indicated by vomiting of blood, low blood pressure, melena, jaundice and gastrointestinal distress [33]. This result shows that there should be a concern for human safety when this plant is used for medicinal purposes. Correlation Table 2 shows that cadmium and copper were positively correlated (r^2^ = 0.671; *p* ≤ 0.05) which indicates these heavy metals are not antagonistic to each other. Addition of cadmium and copper has been shown to severely reduce plant growth as shows by a reduction of total plant biomass, leaf area and basal diameter.

### 2.4. Total Phenolics and Flavonoids

Accumulation of total phenolics and flavonoids in *G. procumbens* was influenced by the treatments (*p* ≤ 0.01; Table 4). Generally, total phenolics and flavonoids was observed to be the highest for Cd 2 (4.21 mg gallic acid/g dry weight) and the lowest for Cd 4 + Cu 140 (1.24 mg gallic acid/g dry weight). Total flavonoids content followed the same trend as total phenolics, where the highest total flavonoids content was observed for Cd 2 which registered 3.21 mg rutin/g dry. The lowest weight was recorded for Cd 4 + Cu 140 which contained only 0.65 mg rutin/g dry weight. The present results are in agreement with the findings of Lakhdar et al. [34] who found tha municipal waste rich in cadmium and copper enhanced the production of total phenolics and flavonoids by 10% and 7% respectively in *Mesembryanthemum edule*. In a previous study conducted by Okem et al. [35] on *Hypoxis hemerocallidera* exposed to cadmium and aluminium they observed the same pattern as our result whereby production of total phenolics and flavonoids was highly reduced when the plant was exposed to a combination of heavy metals. They also found that exposure to lower levels of single heavy metals enhances the production of secondary metabolites. In the present study, the highest production of total phenolics and flavonoids was observed for Cd 2 and Cu 70 i.e., the lowest level of Cd and Cu exposure. The present result suggests that in the current environment where there is a lot of heavy metal contaminants in the soil, the production of secondary metabolites might be reduced due to interactions between these heavy metals. The results imply that *G. procumbens* planted on soil contaminated with heavy metals might show reduced production of secondary metabolites in the plant. Although, the total phenolics and flavonoids was enhanced with increased Cd or Cu application the cultivated *G. procumbens* plants are not safe to be consumed due to the high bioaccumulation of heavy metals that is above the safety levels recommended by the WHO.

### 2.5. Saponin Content

In Table 4 the application of cadmium and copper give significant differences (*p* < 0.05) in the leaf saponin contents in *G. procumbens*. At the end of the experiment, Cd 4 + Cu 140 treatment produced the lowest saponin content (18.75 mg diosgenin equivalent/g DM) compared to 20.45 mg diosgenin equivalent/g DM by Cd 4 + Cu 70, 24.34 mg diosgenin equivalent/g DM by Cd 2 + Cu 140, 28.61 mg diosgenin equivalent/g DM by control, 32.13 mg diosgenin equivalent/g DM by Cd 2 + Cu 70, 40.21 mg diosgenin equivalent/g DM by Cu 140, 45.62 mg diosgenin equivalent/g DM by Cd 4, 50.21 mg diosgenin equivalent/g DM by Cu 70 and 56.71 mg diosgenin equivalent/g DM by Cd 2 treatment. This showed that the total saponin content in the Cd 2, Cd 4, Cu 70 groups was enhanced and it decreased in the Cu 140, Cd 2 + Cu 70, Cd 2 + 140, Cd 4 + Cu 70 and Cd 4 + Cu 140 treatments compared to the control plants. Saponins occur naturally in soybeans, peas, ginseng, herbs, vegetables and yucca [36]. They are phytochemicals, or plant chemicals, possessing detergent qualities that foam when mixed with water. Commercially, saponins are used in beverages and cosmetics as emulsifiers or sweeteners and also fed to livestock to cut down on odour because they bind to ammonia, which contributes to foul smells [37]. It’s believed saponins have a favorable effect on cholesterol levels, can help boost the immune system, have an antioxidant effect, and may even support bone strength [38]. In the current results it was shown that production of diosgenin was reduced when exposed to combination cadmium and copper treatments. Diosgenin is a very important plant phytochemical that possesses medicinal properties, including hypo-cholesterolemic, anti-carcinogenic, anti-inflammatory, antimicrobial and antioxidant activities. This showed the reduction of saponins under combined heavy metal treatments would reduce the medicinal properties of this plant [39].

### 2.6. Antibacterial Activity

The antibacterial activity of *G. procumbens* leaves was tested using the disc diffusion methods at a concentration of 300 µg/disc. The results, show, in Table 5, indicate a significant (*p* < 0.05) inhibitory activity between all of the treatments. The extract showed moderate to appreciable antimicrobial activities against two Gram positive and two Gram negative bacteria. It was found that after single treatment the plant had higher antimicrobial activities compared to the combination treatments in both Gram positive and Gram negative bacteria. The highest antibacterial activity was observed for Cd 2 and Cu 70 treatment and lowest under combination Cd and Cu treatments like Cd 4 + Cu 70 and Cd 4 + Cu 140. In the present study, the highest inhibition zones that ranged from 8.23–9.67 mm in Gram positive and 9.14–9.34 in Gram negative bacteria were obtained after Cd 2 treatments. Nyastatin as standard antimicrobial showed a high inhibition zone at a concentration of 1 µg/disc. The decreased antimicrobial activities under combined treatments of Cd and Cu can be attributed to a decrease in the production of plant secondary metabolites under these conditions. In a recent study, the production of total phenolics, flavonoids and saponin was lowest under combined treatments. Furthermore, total phenolics (r^2^ = 0.901; *p* ≤ 0.05; Table 3), flavonoids (r^2^ = 0.892; *p* ≤ 0.05; Table 3) and saponin content (r^2^ = 0.921; *p* ≤ 0.05; Table 3) had a significant positive correlation with antibacterial activity in the present study. This indicates that the antibacterial activity was influenced by the total phenolics, total flavonoids and saponin content in the plant. A higher secondary metabolites content would enhance the antibacterial activity in *G. procumbens* plant. The same result was also obtained by Okem et al. [35] on *Drimia elata*, where they found the highest antibacterial activity (minimum inhibition concentration; MIC) was observed under single heavy metal treatment (cadmium and aluminium) compared to the combination of Cd and Al. Their other study on *Hypoxis hemerocallidera* [40] also obtained the same result where the combination treatment of Cd and Al produced the lowest secondary metabolites content and this reduced the antimicrobial activity of the plant. It is apparent that in Table 5, Gram positive bacteria have higher sensitivity than Gram negative ones. The reason that Gram-positive bacteria have a higher antibacterial-sensitivity than Gram-negative bacteria could be the differences in their cell membrane constituents [41]. The outer membrane of the Gram-negative cell wall comprises structural lipopolysaccharides, which render the cell wall impermeable to lipophilic solutes, unlike Gram-positive bacteria, which lack this outer membrane [42]. This morphological difference influences their sensitivity to antibacterial agents. The antibacterial activity also was found to be positively correlated with the plant secondary metabolites, so it can be concluded that in the present study a lower production of secondary metabolites under the combination treatment of cadmium and copper would reduce the secondary metabolites content and reduce the antimicrobial activity of *G. procumbens*.

### 2.7. Phenylalanine Ammonia Lyase (PAL) Activity 

**Figure 1:**
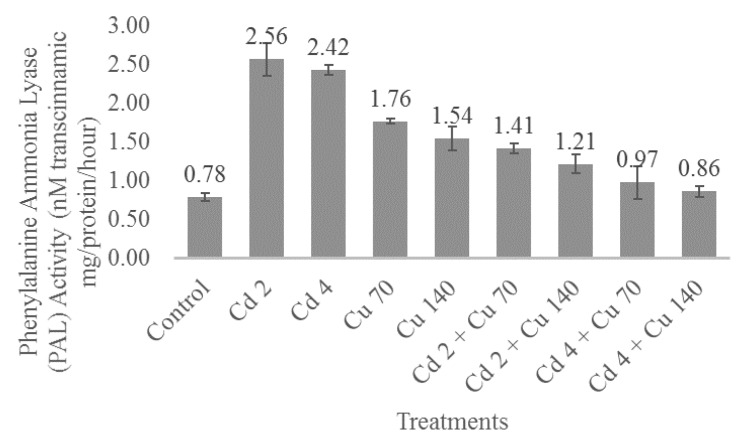
The impact of cadmium and copper on PAL activity of *G. procumbens*. Bars represents standard error of differences between the means. N = 15. Cd 2 = cadmium 2 mg/L; Cd 4 = cadmium 4 mg/L; Cu 70= copper 70 mg/L; Cu 140 = copper 140 mg/L; Cd 2 + Cu 70 = cadmium 2 mg/L + Copper 70 mg/L; Cd 2 + Cu 140 = cadmium 2 mg/L + copper 140 mg/L; Cd 4 + Cu 70 = cadmium 4 mg/L + copper 70 mg/L; Cd 4 + Cu 140 = cadmium 4 mg/L + copper 140 mg/L.

Figure 1 shows the phenylalanine ammonia lyase (PAL) activity was affected (*p* ≤ 0.05) by the heavy metal treatments. In general, it was observed that the mixture of cadmium and copper reduced the activity of this enzyme in *G. procumbens*. The highest PAL activity was found to be under Cd 2 treatment that recorded 2.56 nM *trans*-cinnamic acid mg/protein/h and the lowest was at control, which only registered 0.87 nM *trans*-cinnamic acid mg/protein/h. The increase of PAL activity with increasing rate Cd and Cu might be due to restrictions in protein production due to a limited nitrogen pool under these heavy metal exposure conditions. In the current study, this was shown by reduced total chlorophyll content under these condition that make more phenylalanine available for secondary metabolites production [43]. Correlation analysis revealed that PAL activity had a significant positive correlation with total phenolics (r^2^ = 0.763; *p* ≤ 0.05) total flavonoids (r^2^ = 0.886; *p* ≤ 0.05) and saponin content (r^2^ = 0.811; *p* ≤ 0.05), suggesting when plants are exposed to Cd and Cu, PAL activity would be enhanced and subsequently increase the production of plant secondary metabolites. This was supported by a high and positive correlation coefficient between Cd (r^2^ = 0.776) and Cu (r^2^ = 0.556) with PAL activity that was observed in the present study. The present result was an agreement with the study of Kovacik et al. [44] in *Matricaria chamomilla,* where they observed the stimulation of PAL activity when chamomile was consecutively exposed to excess cadmium (Cd) and copper (Cu) (3, 60, and 120 μM) for seven days.

### 2.8. DPPH Radical Scavenging Assay and Reducing Ability (FRAP Assay)

**Figure 2:**
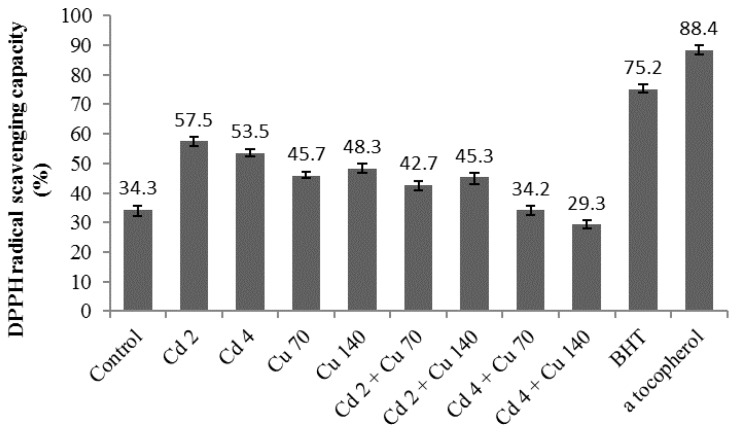
The impact of cadmium and copper on DPPH radical scavenging activity of *G. procumbens*. Bars represents standard error of differences between the means. N = 15. Cd 2 = cadmium 2 mg/L; Cd 4 = cadmium 4 mg/L; Cu 70 = copper 70 mg/L; Cu 140 = copper 140 mg/L; Cd 2 + Cu 70 = cadmium 2 mg/L + copper 70 mg/L; Cd 2 + Cu 140 = cadmium 2 mg/L + copper 140 mg/L; Cd 4 + Cu 70 = cadmium 4 mg/L + copper 70 mg/L; Cd 4 + Cu 140 = cadmium 4 mg/L + copper 140 mg/L.

**Figure 3:**
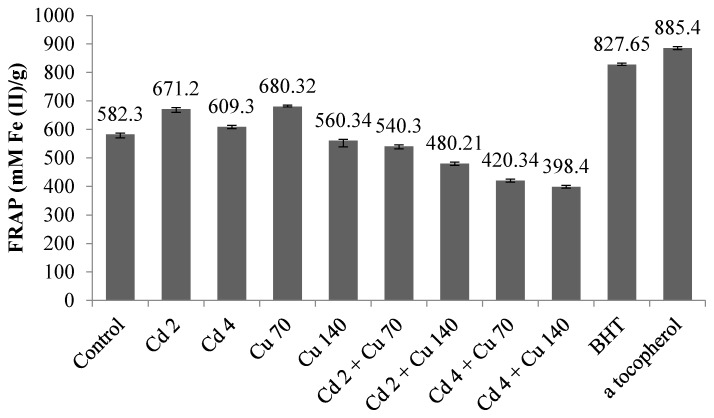
The impact of cadmium and copper on FRAP activity of *G. procumbens*. Bars represents standard error of differences between the means. N = 15. Cd 2 = cadmium 2 mg/L; Cd 4 = cadmium 4 mg/L; Cu 70 = copper 70 mg/L; Cu 140 = copper 140 mg/L; Cd 2 + Cu 70 = cadmium 2 mg/L + copper 70 mg/L; Cd 2 + Cu 140 = cadmium 2 mg/L + copper 140 mg/L; Cd 4 + Cu 70 = cadmium 4 mg/L + copper 70 mg/L; Cd 4 + Cu 140 = cadmium 4 mg/L + copper 140 mg/L.

Figure 2 and Figure 3 show the DPPH and FRAP activity of the leaf extracts of *G. procumbens* under different cadmium and copper treatments. Generally, DPPF and FRAP activity was influenced by the heavy metal treatments (*p* ≤ 0.05). DPPH radical scavenging activity was used to gauge the antioxidant activity of the *G. procumbens* leaf extracts to the heavy metal treatments. DPPH antioxidant activity in the two-heavy metal exposure ranged from 29.2% to 57.5%. Cd 2 treatments showed the highest DPPH activity compared to Cu 70 treatments. With a two-fold increase in Cd and Cu levels the DPPH activity of the plant extracts decreased significantly. The lowest DPPH was observed in Cd 4 + Cu 140 where it just recorded only 29.3%. The treatment extracts showed the lowest DPPH activity compared to positive controls (BHT: 75.4% and α tocopherol: 88.4%). The FRAP value of *G. procumbens* leaves extracts ranged from 398.41–680.32 μM of Fe(II)/g. The highest FRAP activity was observed in Cu 70. Results of the FRAP assay showed that as the rate of cadmium and copper doubled the FRAP activity of leaves extract was reduced significantly. The largest decrease was observed under Cd 4 + Cu 140 treatment that only recorded 398.4 μM of Fe(II). The decrease in antioxidant potential in the leaves under combined heavy metal treatment could be related to the reduction in the content of some phytochemicals, such as total phenolics, total flavonoids and saponin under these conditions. In the present study, there was a positive correlation between total phenolics, flavonoids and saponin content with DPPH and FRAP activity. Positive correlations between level of secondary metabolites and anti-oxidant properties were demonstrated by previous studies [45,46,47,48,49]. Our finding is consistent with previous studies indicating that the levels of secondary metabolites (phenolics and flavonoids, saponin) correspond to the free radical scavenging potential of the medicinal plants [50,51,52,53]. The present study suggests that the antioxidant activity of *G. procumbens* would reduce under combination of cadmium and copper. Although, the antioxidant and antibacterial activities was high under single treatments compared to the combined treatments of Cd and Cu, the plant extractx are not safe to be consumed due to higher Cd and Cu levels than recommended by the WHO observed in the present study.

## 3. Materials and Methods 

### 3.1. Experimental Location, Plant Materials and Treatments 

This experiment was carried out at the Biology Department Greenhouse Complex, Universiti Putra Malaysia. Stem cuttings of *G. procumbens* (Lour.) Merr were propagated for two weeks in small pots and then transferred to pots filled with a soil-less mixture of burnt rice husk and coco peat in the ratio of 3:1. For fertilization, to each pot, 50% Hoaglands nutrient solution was added once a week. Concentrations of heavy metals (100 mL per pot) in the form of CdCl_2_ (2, 4 mg/L); CuSO_4_ (70, 140 mg/L) and combinations of Cd and Cu (Cd 2:Cu 70, Cd 2:Cu 140, Cd 4:Cu 70 and Cd 4:Cu 140) were added once a week until end of experiment. Hoaglands solution (50%) was used for the control treatment. The experiment was terminated after 12 weeks. The experiment was organized in a randomized complete block (RCBD) design with three replications. Each experimental unit contained of five seedlings, and there were a total of 135 seedlings used in the research. The microclimatic condition under the glasshouse are presented in Table 6.

### 3.2. Determination of Plant Biomass, Leaf Area and Basal Diameter

Total plant biomass was taken by mixing the dry weight of root, stems and leaves per seedling. The plant parts were stored in paper bags and oven dried at 80 °C until constant weight was reached. Leaf area per plant was measured using a plant leaf area meter (LI-3100, LICOR Biosciecne, Lincoln, NB, USA). Basal diameter was determined by using Vernier callipers.

### 3.3. Determination of Total Phenolics and Flavonoids 

The method used for extraction and quantification of total phenolics and flavonoids was as described by Ibrahim and Jaafar [52]. Ground leaves tissue samples (0.1 g) were extracted with 80% ethanol (10 mL) on an orbital shaker for 120 min at 50 °C. The mixture was subsequently filtered (Whatman™ No.1, Maidstone, UK), and the filtrate was used for the quantification of total phenolics and total flavonoids. Folin–Ciocalteu reagent (Kanto Chemical, Osaka, Japan; diluted 10-fold) was used to determine the total phenolics content of the leaf samples. Two hundred µL of the sample extract was mixed with Follin–Ciocalteau reagent (1.5 mL) and allowed to stand at 22 °C for 5 min before adding NaNO_3_ solution (Ajax Finechem; New South Wales, Australia, 1.5 mL, 60 g L^−1^). After two hours at 22 °C, absorbance was measured at 725 nm. The results were expressed as mg g^−1^ gallic acid equivalent (Merck, Darmstadt, Germany; mg GAE g^−1^ dry sample). For total flavonoids determination, samples (1 mL) were mixed with NaNO_3_ (0.3 mL) in a test tube covered with aluminium foil and left to stand for 5 min. Then 10% AlCl_3_ (Ajax Finechem; 0.3 mL) was added followed by addition of 1 M NaOH (Ajax Finechem; 2 mL). The absorbance was measured at 510 nm using a spectrophotometer with rutin as a standard (results expressed as mg/g rutin dry sample).

### 3.4. Total Saponin Content

Total saponin content was determined in leaves using vanillin-sulfuric acid colorimetric reaction by Makkar et al. [53]. The standard used for saponin is diosgenin (Merck). 

### 3.5. Cadmium and Copper Analysis

To measure Cd and Cu concentration, one gram of dried plant material samples (shoot and root separately; oven dried at 80 °C) was digested in nitric acid (Kanto Chemical, Tokyo, Japan; HNO_3_; 65%). Cadmium and copper concentration of the extracted solution was measured using an inductively coupled plasma emission (ICP-MS, 7500, Agilent, San Clara, CA, USA).

### 3.6. Chlorophyll Measurement

For the measurement of chlorophyll concentration, weighed fresh leaves (200 mg) were grinded using a mortar and pestle and immersed in 10 mL of 100% acetone. Samples then homogenized with the B-Braun type homogenizer (Labequip, Markam, ON, Canada) at 1000 rpm for one minute. The homogenate was filtered through two layer of cheesecloth, and was centrifuged at 2500 rpm for 10 min. The supernatant was separated and placed in quartz cuvettes and absorbance measured against a blank of 100% acetone at 2 wavelengths. The two wavelengths of 662 nm and 645 nm were used as the peak absorbences of chlorophyll-a and chlorophyll-b. The total amount of chlorophyll-a and chlorophyll-b were then calculated according to the formulas of Lichtentaler and Wellburn [54].

### 3.7. Antibacterial Activity 

Bacterial strains from four species of bacteria (Gram positive and Gram negative) were used. The Gram positive bacteria were *Bacillus subtilis* B 14512 and *Staphylococcus aureus* S15923. The Gram negative bacteria include *Escherichia coli* E 256 and *Klebsiella pneumoniae* K356 that were obtained from the Institute for Medical Research Malaysia. All bacteria were cultured on nutrient agar. The antibacterial assay was performed using disc diffusion (Kirby-Bauer) method. The density of bacteria was standardized using McFarland 0.5 turbidity standard and was swabbed onto Mueller Hinton Agar (Sigma-Aldrich; St. Louis, MO, USA) surface. One mg of leaves crude extract was dissolved initially in 100 μL methanol and loaded onto sterile Whatman No. 1 filter paper discs (6 mm diameter) and the discs were impregnated onto inoculated agar. The plates were left at 4 °C for an hour to allow the diffusion of extracts before they were incubated for 16–20 h at 37 °C. Antibacterial activities was indicated when clear inhibition zones were noted around the discs. Nyastatin (Oxoid; Chicago, IL, USA) at 10 μg/mL was used as a positive control. The diameter of the inhibition zones was measured and the results were expressed as mean of three independent experiments. The test was repeated three times [55].

### 3.8. Phenylalanine Ammonia-Lyase (PAL) Activity 

Phenylalanine-ammonia-lyase (PAL) activity was measured using the method described by Martinez and Lafuante [56]. The enzyme activity was determined by spectrophotometrically measuring the production of trans-cinnamic acid from l-phenylalanine. Enzyme extract (10 μL) was incubated at 40 °C with 12.1 mM l-phenylalanine (90 μL, Sigma; St Louis, MS, USA) that was prepared in 50 mM Tris-HCl, (pH 8.5). After 15 min of reaction, trans-cinnamic acid yield was estimated by measuring increase in the absorbance at 290 nm. The standard curve was prepared by using a trans-cinnamic acid standard (Sigma) and the PAL activity was expressed as nM trans-cinnamic acid/μg protein/h.

### 3.9. DPPH Radical Scavenging Assay

The DPPH free radical scavenging activity of each sample was determined according to the method described by Mensor et al. [57]. A solution of 0.1 mM DPPH in methanol was prepared. The initial absorbance of the DPPH in methanol was measured at 515 nm. An aliquot (40 µL) of an extract was added to 3 mL of methanolic DPPH solution. The change in absorbance at 515 nm was measured after 30 min. The antiradical activity (AA) was determined using the following formula: AA% = 100 − [(Abs: sample − Abs: empty sample)] × 100)/Abs: control the optic density of the samples, the control and the empty samples were measured in comparison with methanol. A synthetic antioxidant, BHT (butylated hydroxytoluene; Sigma) and α-tocopherol (Sigma), were used as positive controls.

### 3.10. Reducing Ability (FRAP Assay) 

The ability to reduce ferric ions was measured using modifying methods of Benzie and Strain [58]. An aliquot (200 µL) of the extract with appropriate dilution was added to 3 mL of FRAP reagent (Ajax, Berlin, Germany; 10 parts of 300 mM sodium acetate buffer (Sigma) at pH 3.6, 1 part of 10 mM TPTZ solution and 1 part of 20 mM FeCl_3_·6H_2_O solution) and the reaction mixture was incubated in a water bath at 37 °C. The increase in absorbance at 593 nm was measured after 30 min. The antioxidant capacity based on the ability to reduce ferric ions of the extract was expressed as expressed in µM Fe(II)/g dry mass and compared with those of standards for BHT (Sigma), ascorbic acid (Sigma), and α-tocopherol (Ajax). 

### 3.11. Statistical Analysis

Data were analysed using analysis of variance using SAS version 17 (SAS institute, Carry, NC, USA). Mean separation test between treatments was performed using Duncan multiple range test and standard error of differences between means was calculated with the assumption that data were normally distributed and equally replicated.

## 4. Conclusions

This study was devoted to determining the effects of Cd and Cu on the growth, heavy metal bioaccumulation and biochemical changes in the medicinal plant *G. procumbens* to predict the effect on the efficacy of this plant. It was found that combined Cd and Cu treatment had the greatest influence on the growth, heavy metal accumulation and biochemical changes in *G. procumbens*. Cadmium and copper exposure reduced the plant growth which was indicated by a reduction in total plant dry weight, leaf area and basal diameter. The reduction of total chlorophyll content was observed in all treatments except control indicating that *G. procumbens* was under stress conditions when exposed to the heavy metals. The accumulation of heavy metals in shoot and root was higher than the rate recommended by the WHO or consumer exposure to heavy metal contamination when consumed. The combined heavy metal exposure (Cd + Cu) has shown to reduce the efficacy of this herbal plant, reducing the production of total phenolics, total flavonoids, saponins, antibacterial, DPPH and FRAP activity compared to single exposure to Cd or Cu. Although the antioxidant and antimicrobial activity was highest under single heavy metal treatment, the heavy metal content of Cd and Cu exceeded the safety level recommended by the WHO indicating that *G. procumbens* planted in contaminated soil is not safe for consumption. In future work the study of the interaction effects of other heavy metals such as lead, zinc, and chromium is suggested to understand the impact of these heavy metal interactions on the efficacy of medicinal plants.

## Figures and Tables

**Table 1:** Plant dry weight, total leaf area, basal diameter, chlorophyll properties of *Gynura procumbens* to different cadmium and copper treatments.

Treatments	Plant Dry Weight (g)	Total Leaf Area (cm^2^)	Basal Diameter (mm)	Chlorophyll a (mg/g Fresh Weight)	Chlorophyll b (mg/g Fresh Weight)	Chlorophyll a + b	Chlorophyll a/b
Control	32.45 ± 1.23 ^a^	425.32 ± 12.21 ^a^	24.12 ± 2.34 ^a^	27.52 ± 1.23 ^a^	32.15 ± 0.23 ^a^	59.32 ± 6.76 ^a^	0.84 ± 0.09 ^a^
Cd 2	27.45 ± 0.34 ^b^	397.56 ± 10.23 ^b^	19.32 ± 0.98 ^b^	20.14 ± 0.54 ^b^	28.76 ± 0.76 ^b^	47.12 ± 3.32 ^b^	0.71 ± 0.02 ^b^
Cd 4	23.34 ± 2.32 ^c^	365.21 ± 8.67 ^d^	17.25 ± 2.34 ^c^	19.21 ± 2.34 ^b^	26.54 ± 0.21 ^c^	45.23 ± 2.45 ^c^	0.73 ± 0.01 ^b^
Cu 70	24.21 ± 1.45 ^c^	387.21 ± 9.21 ^c^	18.72 ± 4.32 ^b^	18.23 ± 2.34 ^c^	27.54 ± 0.34 ^c^	45.11 ± 3.12 ^c^	0.67 ± 0.07 ^c^
Cu 140	20.17 ± 2.45 ^d^	340.17 ± 6.23 ^e^	15.21 ± 2.45 ^e^	16.21 ± 1.32 ^d^	25.43 ± 1.34 ^d^	41.32 ± 5.12 ^d^	0.64 ± 0.03 ^c^
Cd 2 + Cu 70	17.56 ± 1.21 ^e^	320.12 ± 7.34 ^f^	17.21 ± 1.43 ^c^	13.21 ± 2.31 ^e^	24.56 ± 1.45 ^e^	37.21 ± 0.23 ^e^	0.54 ± 0.02 ^d^
Cd 2 + Cu 140	15.32 ± 0.21 ^f^	301.23 ± 5.64 ^g^	16.32 ± 3.23 ^d^	11.23 ± 1.21 ^f^	23.21 ± 2.34 ^f^	34.21 ± 0.78 ^f^	0.47 ± 0.04 ^e^
Cd 4 + Cu 70	12.31 ± 1.23 ^g^	311.24 ± 2.45 ^h^	13.21 ± 3.12 ^f^	11.67 ± 2.14 ^f^	23.44 ± 4.22 ^f^	33.23 ± 0.98 ^f^	0.48 ± 0.07 ^e^
Cd 4 + Cu 140	12.52 ± 1.23 ^g^	290.32 ± 3.45 ^i^	12.12 ± 0.23 ^g^	10.21 ± 4.21 ^g^	21.23 ± 2.45 ^g^	30.34 ± 1.23 ^g^	0.47 ± 0.05 ^e^

All analyses are mean ± standard error of mean (SEM), N = 15. Means not sharing a common single letter were significantly different at *p* ≤ 0.05 using Duncan Multiple Range Test (DNMRT). Cd 2 = cadmium 2 mg/L; Cd 4 = cadmium 4 mg/L; Cu 70 = copper 70 mg/L; Cu 140 = copper 140 mg/L; Cd 2 + Cu 70 = cadmium 2 mg/L + copper 70 mg/L; Cd 2 + Cu 140 = cadmium 2 mg/L + copper 140 mg/L; Cd 4 + Cu 70 = cadmium 4 mg/L + copper 70 mg/L; Cd 4 + Cu 140 = cadmium 4 mg/L + copper 140 mg/L.

**Table 2:** Correlations among the measured parameters in the experiments.

Parameters	1	2	3	4	5	6	7	8	9	10	11	12	13
1. T. Biomass	1.000												
2. Area	0.321	1.000											
3. Basal	0.223	0.213	1.000										
4. T.Chlorophyll	0.432	0.432	0.211	1.000									
5. Cadmium	−0.671 *	−0.321	−0.567 *	−0.765 *	1.000								
6. Copper	−0.621 *	−0.561 *	−0.444	0.432	0.671 *	1.000							
7. T.phenolics	0.213	0.213	0.231	−0.871 *	0.761 *	0.661 *	1.000						
8. T flavonoids	0.098	0.123	0.116	−0.712 *	0.665	0.687 *	0.876 *	1.000					
9. Saponins	0.321	0.091	0.092	−0.812 *	0.521	0.622 *	0.812 *	0.776 *	1.000				
10. PAL activity	0.021	0.034	0.112	0.321 *	0.776 *	0.556	0.763 *	0.886 *	0.811 *	1.000			
11. DPPH	0.231	0.091	0.121	0.221	0.776 *	0.453	0.879 *	0.910 *	0.762 *	0.891 *	1.000		
12. FRAP	0.121	0.213	0.005	0.234	0.662	0.321	0.912 *	0.927 *	0.874 *	0.932 *	0.782 *	1.000	
13. Antibacterial	0.123	0.217	0.321	0.453	0.091	0.231	0.901 *	0.892 *	0.921 *	0.875 *	0.862 *	0.877 *	1.000

* Significant at *p* ≤ 0.05.

**Table 3:** Uptake and translocation factor (TF) of Cd and Cu in *Gynura procumbens* roots and shoot under different cadmium and copper treatments. ND = not detected.

Treatments	Cd Concentration (mg/kg)			Cu Concentration (mg/kg)		
	Root	Shoot	TF	Root	Shoot	TF
Control	ND	ND	ND	ND	ND	ND
Cd 2	1.21 ± 0.21 ^e^	0.76 ± 0.09 ^d^	0.62	ND	ND	ND
Cd 4	2.31 ± 0.16 ^b^	1.21 ± 0.01 ^b^	0.52	ND	ND	ND
Cu 70	ND	ND	ND	35.61 ± 1.23 ^c^	24.43 ± 0.12 ^d^	0.68
Cu 140	ND	ND	ND	66.43 ± 0.76 ^b^	37.45 ± 1.34 ^b^	0.56
Cd 2 + Cu 70	1.17 ± 0.21 ^e^	1.32 ± 0.21 ^a^	1.12	34.56 ± 1.45 ^d^	32.12 ± 0.76 ^c^	0.94
Cd 2 + Cu 140	1.45 ± 0.09 ^d^	1.43 ± 0.17 ^a^	0.98	75.23 ± 2.34 ^a^	54.16 ± 1.23 ^a^	0.71
Cd 4 + Cu 70	2.65 ± 0.21 ^a^	1.26 ± 0.11 ^b^	0.47	38.32 ± 1.63 ^c^	32.12 ± 0.34 ^c^	0.83
Cd 4 + Cu 140	2.15 ± 0.42 ^c^	0.98 ± 0.12 ^c^	0..45	72.31 ± 1.21 ^a^	52.34 ± 1.45 ^a^	0.72

All analyses are mean ± standard error of mean (SEM), N = 15. Means not sharing a common single letter were significantly different at *p* ≤ 0.05 using Duncan Multiple Range Test (DNMRT). Cd 2 = cadmium 2 mg/L; Cd 4 = cadmium 4 mg/L; Cu 70 = copper 70 mg/L; Cu 140 = copper 140 mg/L; Cd 2 + Cu 70 = cadmium 2 mg/L + copper 70 mg/L; Cd 2 + Cu 140 = cadmium 2 mg/L + copper 140 mg/L; Cd 4 + Cu 70 = cadmium 4 mg/L + copper 70 mg/L; Cd 4 + Cu 140 = cadmium 4 mg/L + copper 140 mg/L.

**Table 4:** Total phenolics, total flavonoids and total saponin content of *Gyanura procumbens* under different cadmium and copper treatments.

Treatments (mg/L)	Total Phenolics (mg/g DM GAE )	Total Flavonoids (mg/g DM Rutin)	Total Saponin (mg/g DM Diosgenin)
Control	2.15 ± 0.15 ^e^	1.25 ± 0.13 ^e^	28.61 ± 1.23 ^g^
Cd 2	4.21 ± 0.21 ^a^	3.21 ± 0.14 ^a^	56.71 ± 2.34 ^a^
Cd 4	3.21 ± 0.14 ^c^	2.76 ± 0.67 ^b^	45.62 ± 1.13 ^c^
Cu 70	4.02 ± 0.21 ^a^	2.87 ± 0.45 ^b^	50.21 ± 2.45 ^b^
Cu 140	3.76 ± 0.54 ^b^	2.45 ± 0.27 ^c^	40.21 ± 2.12 ^d^
Cd 2 + Cu 70	2.56 ± 0.21 ^d^	2.21 ± 0.13 ^c^	32.13 ± 3.21 ^e^
Cd 2 + Cu 140	1.78 ± 0.32 ^f^	1.76 ± 0.09 ^d^	24.34 ± 1.76 ^f^
Cd 4 + Cu 70	1.65 ± 0.15 ^f^	0.98 ± 0.13 ^f^	20.45 ± 1.21 ^h^
Cd 4 + Cu 140	1.24 ± 0.14 ^g^	0.65 ± 0.17 ^g^	18.75 ± 1.76 ^i^

All analyses are mean ± standard error of mean (SEM), N = 15. Means not sharing a common single letter were significantly different at *p* ≤ 0.05 using Duncan Multiple Range Test (DNMRT). Cd 2 = cadmium 2 mg/L; Cd 4 = cadmium 4 mg/L; Cu 70 = copper 70 mg/L; Cu 140 = copper 140 mg/L; Cd 2 + Cu 70 = cadmium 2 mg/L + copper 70 mg/L; Cd 2 + Cu 140 = cadmium 2 mg/L + copper 140 mg/L; Cd 4 + Cu 70 = cadmium 4 mg/L + copper 70 mg/L; Cd 4 + Cu 140 = cadmium 4 mg/L + copper 140 mg/L.

**Table 5:** Antimicrobial activity of *Gynura procumbens* leaves crude extracts of different cadmium and copper treatments based on disc diffusion method.

Treatments	Inhibition Zone (mm)			
	Gram Positive Bacteria		Gram Negative Bacteria	
	*B. subtilis*	*S. aureus*	*E. coli*	*K. pneumonie*
Control	7.65 ± 0.11 ^d^	6.32 ± 0.11 ^f^	7.26 ± 0.12 ^d^	8.88 ± 0.21 ^c^
Cd 2	9.67 ± 0.14 ^b^	8.23 ± 0.21 ^b^	9.34 ± 0.09 ^b^	9.25 ± 0.23 ^b^
Cd 4	8.21 ± 0.21 ^c^	7.32 ± 0.14 ^c^	8.78 ± 0.23 ^c^	8.23 ± 0.15 ^d^
Cu 70	9.11 ± 0.09 ^b^	8.12 ± 0.12 ^b^	9.12 ± 0.45 ^b^	9.06 ± 0.12 ^b c^
Cu 140	8.03 ± 0.23 ^c^	7.21 ± 0.21 ^c^	8.26 ± 0.12 ^c^	8.17 ± 0.12 ^d^
Cd 2 + Cu 70	7.34 ± 0.11 ^d^	7.02 ± 0.12 ^d^	7.23 ± 0.45 ^d^	7.45 ± 0.11 ^e^
Cd 2 + Cu 140	7.21 ± 0.21 ^e^	6.98 ± 0.21 ^e^	7.02 ± 0.67 ^e^	7.56 ± 0.13 ^e^
Cd 4 + Cu 70	6.12 ± 0.23 ^f^	6.56 ± 0.14 ^f^	6.78 ± 0.21 ^f^	6.42 ± 0.13 ^f^
Cd 4 + Cu 140	6.56 ± 0.12 ^f^	6.09 ± 0.21 ^g^	6.56 ± 0.16 ^f^	6.01 ± 0.23 ^f^
Nyastatin *	12.12 ± 0.14 ^a^	11.21 ± 0.37 ^a^	13.54 ± 0.31 ^a^	11.45 ± 0.16 ^a^

* At 100 μg/mL. All analyses are mean ± standard error of mean (SEM), N = 15. Means not sharing a common single letter were significantly different at *p* ≤ 0.05 using Duncan Multiple Range Test (DNMRT). Cd 2 = cadmium 2 mg/L; Cd 4 = cadmium 4 mg/L; Cu 70 = copper 70 mg/L; Cu 140 = copper 140 mg/L; Cd 2 + Cu 70 = cadmium 2 mg/L + copper 70 mg/L; Cd 2 + Cu 140 = cadmium 2 mg/L + copper 140 mg/L; Cd 4 + Cu 70 = cadmium 4 mg/L + copper 70 mg/L; Cd 4 + Cu 140 = cadmium 4 mg/L + copper 140 mg/L.

**Table 6:** Microclimatic condition under the research area during experiments.

Microclimate Parameters	Quantification
Relative humidity	57.25–66.34%
Light intensity	120–1630 μmol/m^2^/s
Day temperature	29–34 °C
Night temperature	18–23 °C
Ambient CO_2_	382.56 μmol/mol
